# FKF1 Interacts with CHUP1 and Regulates Chloroplast Movement in Arabidopsis

**DOI:** 10.3390/plants12030542

**Published:** 2023-01-25

**Authors:** Ning Yuan, Lavanya Mendu, Kaushik Ghose, Carlie Shea Witte, Julia Frugoli, Venugopal Mendu

**Affiliations:** 1 Department of Plant and Soil Science, Texas Tech University, Lubbock, TX 79409, USA; 2 Department of Plant Sciences and Plant Pathology, Montana State University, Bozeman, MT 59717, USA; 3 Department of Genetics & Biochemistry, Clemson University, Clemson, SC 29634, USA

**Keywords:** *Arabidopsis thaliana*, avoidance response, blue light, chloroplast movement, light receptor, photorelocation

## Abstract

Plants have mechanisms to relocate chloroplasts based on light intensities in order to maximize photosynthesis and reduce photodamage. Under low light, chloroplasts move to the periclinal walls to increase photosynthesis (accumulation) and move to the anticlinal walls under high light to avoid photodamage, and even cell death (avoidance). Arabidopsis blue light receptors phot1 and phot2 (phototropins) have been reported to regulate chloroplast movement. This study discovered that another blue light receptor, FLAVIN-BINDING KELCH REPEAT F-BOX1 (FKF1), regulates chloroplast photorelocation by physically interacting with chloroplast unusual positioning protein 1 (CHUP1), a critical component of the chloroplast motility system. Leaf cross-sectioning and red-light transmittance results showed that overexpression of FKF1 compromised the avoidance response, while the absence of FKF1 enhanced chloroplast movements under high light. Western blot analysis showed that CHUP1 protein abundance is altered in FKF1 mutants and overexpression lines, indicating a potential regulation of CHUP1 by FKF1. qPCR results showed that two photorelocation pathway genes, JAC1 and THRUMIN1, were upregulated in FKF1-OE lines, and overexpression of FKF1 in the *THRUMIN1* mutant weakened its accumulation and avoidance responses, indicating that JAC1 and THRUMIN1 may play a role in the FKF1-mediated chloroplast avoidance response. However, the precise functional roles of JAC1 and THRUMIN1 in this process are not known.

## 1. Introduction

Light is essential for plant photosynthesis, photomorphogenesis, cellulose biosynthesis, and flowering time regulation [1,2,3], but high intensity light causes photo damage to the chloroplasts, and even cell death in plants [4]. Photorelocation of chloroplasts in response to variable natural light conditions confers plants with the ability to maximize photon capture for photosynthesis under low light and avoid photodamage under high light [5,6]. Under dark conditions, chloroplasts are located at the bottom wall of the palisade cells [6]; however, chloroplasts move to different positions in response to variations in light intensity. Chloroplasts accumulate on the periclinal (upper) wall of palisade cells to increase the photosynthetic efficiency under low light (accumulation response). In contrast, chloroplasts assemble on the anticlinal (side) wall of palisade cells to avoid photodamage under high light (avoidance response) [5,6,7]. Of the seven characterized blue light/UV light receptors (phot1, phot2, Cry1, Cry2, FKF1, ZTL, and LKP) in *Arabidopsis thaliana* [8], phot1 (phototropin1) and phot2 (phototropin2) have been reported to mediate the photorelocation of chloroplasts [9,10,11,12]. While both proteins are involved in the accumulation response, only phot2 mediates the avoidance response [13,14], indicating that the two phototropins appear to have different functions in chloroplast photorelocation movements. Phototropins are comprised of two N-terminal LOV (light, oxygen, and voltage) domains and a C-terminal serine/threonine kinase domain [15]. Perception of blue light by the LOV domains induces the conformational change in the C-terminal kinase domain, permitting phot1/2 to phosphorylate the downstream components of the blue light signaling pathway, such as BLUS1, phytochrome kinase substrate 4, and others [16,17,18].

Light induced chloroplast movement is also observed in other green plant species such as green algae (*Mougeotia scalaris*), mosses (*Physcomitrella patens*), and ferns (*Adiantum capillus-veneris*). Corresponding phototropin-like genes have been cloned from these plants and then characterized [13,19,20,21]. Acphot2 mediates the blue light induced avoidance response in *A. capillus-veneris*, and four PpPhots (PHOTA1, PHOTA2, PHOTB1, and PHOTB2) are responsible for the light induced chloroplast movement in *P. patens* [19,20]. Unlike angiosperms, which exhibit only blue light induced chloroplast movement, cryptogam plants also utilize red light as a stimulus to regulate the chloroplast photorelocation [13,22,23,24,25]. NEO1 (neochrome1), a specific dual-light photoreceptor in *A. capillus-veneris,* mediates the chloroplast accumulation movement in *A. capillus-veneris* in response to red as well as blue light [26]. NEO1 is a chimeric protein comprised of the N-terminal PHY photosensory domain of phytochromes and a complete phototropin in the C-terminal [26]. Two photoreceptors with structures similar to AcNEO1 were later identified in green algae *M. scalaris*; however, MsNEO1 and MsNEO2 do not absorb UV/blue light. This might be due to the less conserved FMN interaction or photocycle between the LOV domains of AcNEO1 and MsNEO1/2 [13,21]. Complementation of the *A. capillus-veneris* neo1 mutant with *MsNEO1* and *MsNEO2* genes rescued the red light mediated chloroplast movements of neo1, indicating that Msneo1 and Msneo2 are two functional red light receptors [21]. There are no identified homologs in *P. patens*, which instead has four phototropins that function downstream of the phytochromes in the red light signaling pathway. Thus, phytochromes and phototropins together regulate the chloroplast movement under both red and blue light conditions in the cryptogamae plant *P. patens* [19].

Spermatophytes constantly monitor the variations in light intensities through phototropins (phot1 and phot2) for chloroplast accumulation and avoidance responses [27]. The time lag in responding to light in Arabidopsis and fern *A. capillus-veneris* suggests that accumulation and avoidance responses may depend on signaling cascades [28,29,30]. Several key intermediate players in the downstream signaling pathway have been identified and characterized [31,32,33]. JAC1 (J-domain protein required for chloroplast accumulation response 1) is involved in the light mediated accumulation response downstream of PMI1 (plastid movement impaired 1), which takes part both in accumulation and avoidance responses, while PMI2 (plastid movement impaired 2) is exclusively involved in the avoidance response. In addition, WEB1 (weak chloroplast movement under blue light 1), identified as a repressor of JAC1, is also involved in the avoidance response [33,34,35,36,37]. A recent study suggests that ROS (reactive oxygen species), particularly, H_2_O_2_, act as the immediate cellular products of high light irradiance and could possibly be the earliest signaling molecules to induce the avoidance response [38]. The ROS mediated avoidance response is possibly triggered through the ROS-Ca^2+^-cp-actin communication system and the NOX (NADPH oxidase)-plasma membrane H^+^-ATPase positive feed-forward loop [38]. In addition, it has been suggested that the levels of cytosolic Ca^2+^ and phosphatidylinositol lipid are involved in mediating signal transduction for the photorelocation movement of chloroplasts [39]. In addition, the inactivation of 4,5-bisphosphate [PI-4-5-P2]-PLC, a calcium mobilizing pathway, was able to suppress only the phot2 mediated accumulation and avoidance responses. However, the inactivation of phosphatidylinositol 4-kinase (PI4K) and the 3-kinase (PI3K) mediated cytosolic Ca^2+^ concentration regulation pathway strongly repressed both the phot1 and phot2 mediated accumulation response, attributed to the disrupted Ca^2+^ signaling [39]. Identification of these critical components in the signaling pathway sheds light on the precise understanding of the timely response of the photorelocation signal transduction pathways.

While the light mediated chloroplast relocation signal transduction pathway is still a mystery, the chloroplast motility system involved in the physical movement of the chloroplast itself is also not very well understood. The CHUP1 (chloroplast unusual positioning protein 1) is a key protein in the chloroplast motility system which physically links chloroplasts, actin-filaments, and the plasma membrane to facilitate chloroplast movement [31,40,41]. CHUP1 was first identified in *Arabidopsis thaliana*, and its orthologs showing highly conserved structures were identified in the cryptogam *Physcomitrella patens* [31,42]. Both AtCHUP1 and PpCHUP1 mediate the actin-dependent light induced avoidance response [31,42]. In Arabidopsis, the N-terminus of CHUP1 is inserted into the outer envelope of the chloroplast, while its coiled-coil region binds to the plasma membrane on the anticlinal walls [31]. The movement of chloroplasts on the plasma membrane depends on specialized actin filaments known as chloroplast-actin filaments (cp-actin) [41]. The actin-binding domain of CHUP1 interacts with cp-actin, regardless of its filamentous state, while the movement of the chloroplast is consistent with the filamentous state [41,43]. Under high light, cp-actin filaments depolymerize in the posterior region of the chloroplast, but rapidly polymerize in the anterior region, which generates a traction force driving the directional movement of chloroplasts towards the anticlinal wall [43]. Under low light or dark conditions, the cp-actin filaments rearrange in all directions of the chloroplast, leading to the distribution of chloroplasts on the periclinal wall, as well as at the bottom of the palisade cells [16]. Further, THRUMIN1 also binds and regulates cp-actin reorganization at the plasma membrane [32]. Two kinesin-like protein-coding genes, KAC1 and KAC2, mediate the anchoring and movement of chloroplasts on the plasma membrane [32,44].

Seasonal day length changes are sensed by plant photoreceptors at a specific time during the day for flowering time regulation [45]. This regulation requires transcription of FLOWERING LOCUS T (FT) triggered by transcription factor CONSTANS (CO) to induce photoperiodic flowering time in *Arabidopsis* [46,47,48,49]. FKF1 is known to interact with CO through its LOV DOMAIN and stabilizes CO only in the afternoon of long days, while CO is degraded in the dark by the proteasome [46,50]. Further, FKF1 represses CYCLING DOF FACTOR 1 (CDF1), which is a repressor of CO and FT transcription. FKF1 mediates photoperiodic flowering, not only by the activation of the CO diurnal peaks in a light dependent manner to induce FT transcription, but also by the suppression of the FT repressor CDF1 during long-day afternoons [51]. The FKF1 mRNA levels fluctuate in a circadian rhythm [3], and the oscillation is critical for Arabidopsis to differentiate diurnal changes from circadian clock functions [52]. Additionally, blue light receptors are involved in rapid reversible movements, such as chloroplast movements, in response to changes in light intensity and light induced stomatal movements [15,53]. Recent studies showed the role of FKF1 in cellulose synthesis [1], and the present study establishes the function of FKF1 in chloroplast movement.

This study establishes the role of FLAVIN-BINDING KELCH REPEAT F-BOX1 (FKF1) in the photorelocation of chloroplasts through direct physical interaction with and regulation of the CHUP1 protein. Our data show that FKF1 physically interacts with the C-terminus of CHUP1 and regulates chloroplast movement. Red-light transmittance experiments show that the avoidance response is boosted in the FKF1 mutant, while it is compromised in FKF1 overexpression lines. The over-expression of *FKF1* leads to an unusual distribution of chloroplasts in the palisade cells under high light. Our data also show that the protein level of CHUP1 might be regulated by FKF1 protein abundance, and the expression of *JAC1* and *THRUMIN1* increased in *FKF1* overexpression lines through an unknown mechanism. Overall, FKF1, previously reported to regulate photoperiodic flowering time and cellulose biosynthesis, is now also known to be involved in the regulation of the photorelocation movement of chloroplasts, in addition to phot1 and phot2.

## 2. Results

### 2.1. FKF1 Physically Interacts with CHUP1

**Figure 1:**
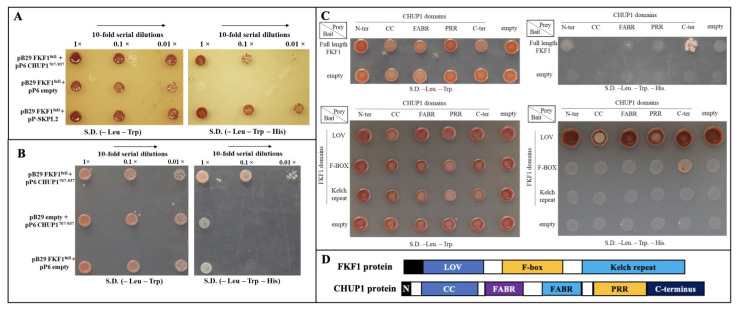
FKF1 physically interacts with CHUP1 protein. (**A**) FKF1 protein shows interaction with CHUP1^707–937^ in a yeast two-hybrid assay. Full length FKF1 cDNA was cloned into the pB29 bait vector and cDNA encoding CHUP1^707–937^ was cloned into the pP6 prey vector and transformed into yeast strain TATA. The pP6 empty prey vector and pP6-SKPL2 were used as negative and positive controls, respectively. (**B**) CHUP1^707–937^ did not interact with the empty bait vector. To rule out the possibility of CHUP1^707–937^ interaction with the empty vector, cDNA encoding CHUP1^707–937^ was cloned into the pP6 prey vector, and the interaction with pB29 empty vector was tested. (**C**) The F-BOX domain of FKF1 interacts with the C-terminal domain of CHUP1^707–937^. The upper panel shows the FKF interaction with the C-terminal domain of CHUP1. Full length FKF1 was cloned into the pB29 vector, and cDNAs encoding CHUP1 domains were cloned into the pP6 vector and tested for interactions. The lower panel shows CHUP1 C-terminal interaction with the F-BOX domain of FKF1. Different domains of FKF1 were cloned into the pB29 vector, and different domains of CHUP1 were cloned into the pP6 prey vector and tested for domain interactions. (**D**) Protein domain organization of FKF1 and CHUP1. LOV: light-oxygen-voltage-sensitive domain. N: N-terminus; CC: coiled-coil domain; FABR: F-actin binding region; PRR: pro-rich region. Yeast media used for interactions: Two selection media are used. First selection media –LW.(–Leu.–Trp) media lacks both leucine and tryptophan in which yeast harboring both prey and bait vectors survive. The second selection media –HLW (–Leu.–Trp.–His.) lacks leucine, tryptophan, and histidine, and selects yeast with both prey and bait vectors that interact.

Genome-wide protein–protein interaction studies (ULTImate Y2H™, Hybrigenics Services, S.A.S., Evry, France) were used to determine that the blue light receptor FKF1 interacts with CHUP1. The FKF1 protein (aa1–aa619) was used as a bait to screen a genome-wide library of one-week-old Arabidopsis seedlings, and the results showed an interaction of FKF1 with truncated CHUP1 (aa707–aa937). To further confirm the genome-wide screening results, full-length FKF1 cDNA was cloned into the pB29 bait vector, while independently, full length CHUP1 protein (aa1–aa1004) and its C-terminal region (aa707–aa937) were cloned into the pP6 prey vectors. Since our yeast two-hybrid results, as well as published evidence [54], showed that FKF1 strongly interacts with the SKP2 (SKP-Like 2) protein, the interaction between FKF1 and SKPL2 was used as a positive control. Bait and prey constructs were transformed into the MATAa yeast strain (+ade2-101:loxP-kanMX-loxP). The full length CHUP1 did not show any interactions with the FKF1 protein, as it has a transmembrane domain that interferes with the Y2H system (Figure S1). Positive interactions were observed for pB29-FKF1/pP6-CHUP1^aa707–937^ and pB29-FKF1/pP6-SKP-L2 (+ve control), while no interaction was observed in the negative control pB29-FKF1/pP6-empty vector (Figure 1A). To exclude the possibility of a DNA binding domain (DBD) LexA interaction in the bait vector pB29 with the GAL4-CHUP1^aa707–937^ fusion protein, we tested the interaction between pB29-empty and pP6-CHUP1^aa707–937^ and observed no interaction (Figure 1B), confirming that FKF1 and CHUP1 are physically interacting.

### 2.2. F-Box Domain of FKF1 Interacts with the C-Terminal Domain of CHUP1

CHUP1 contains multiple functional domains; to identify the specific CHUP1 domain interacting with FKF1, the N-terminal (aa1–aa57), CC (coiled-coil) domain (aa53–aa283), FABR (F-actin binding region) domain (aa311–416), Pro-Rich Region domain (aa623–aa717), and C-terminal (aa707–aa1004) of CHUP1 were cloned separately into the pP6 prey vector and tested for interactions with FKF1 full length protein. The results showed that only the C-terminal domain of CHUP1 interacts with FKF1 (Figure 1C, upper panel). We further tested the specific FKF1 functional domain that is interacting with the CHUP1 protein. The LOV domain (aa50–aa147), F-box (aa204–aa263), and Kelch repeat (aa250–aa619) domains of FKF1 were cloned into the pB29 bait vector separately and tested for interactions with different domains of CHUP1. The results show that the F-box of FKF1 interacts with the C-terminal domain of CHUP1 (Figure 1C, lower panel).

The interaction between full length FKF1 and the C-terminal of CHUP1^aa707–937^ was confirmed using a yeast two-hybrid system. However, because the interaction between FKF1 and full length CHUP1 was not observed in yeast due to the presence of the transmembrane domain (Figure S1), a different approach was taken. To test the full length interaction, a BiFC (bimolecular fluorescence complementation) assay was performed [55] with full-length FKF1 and CHUP1 proteins. Two constructs were made by fusing the two split domains of the Venus protein with FKF1 and CHUP1. The first construct was made by fusing the full length FKF1 to the N-terminal domain (VYNE) of the Venus protein, while the second construct was made by fusing the full-length CHUP1 protein to the C-terminal domain (VYCE) of the Venus protein. The CERK1-VYCE/CERK1-VYNE, which showed a strong interaction in BiFC [56,57], was used as a positive control, while the empty-VYCE/empty-VYNE, was used as a negative control. Transient expression analysis in *Nicotiana benthamiana* leaves (Figure S2) showed a strong interaction between FKF1 and CHUP1 (CHUP1-VYCE/FKF1-VYNE) and the positive control (CERK1-VYCE/CERK1-VYNE), while there was no interaction in the empty vector control (empty-VYCE/empty-VYNE). Thus, both BiFC and Y2H data confirm that FKF1 and CHUP1 interact with each other in vivo and suggest that FKF1 could play an important role in chloroplast movement through physical interaction.

### 2.3. Overexpression of FKF1 Alters the Chloroplast Localization within the Cells

**Figure 2:**
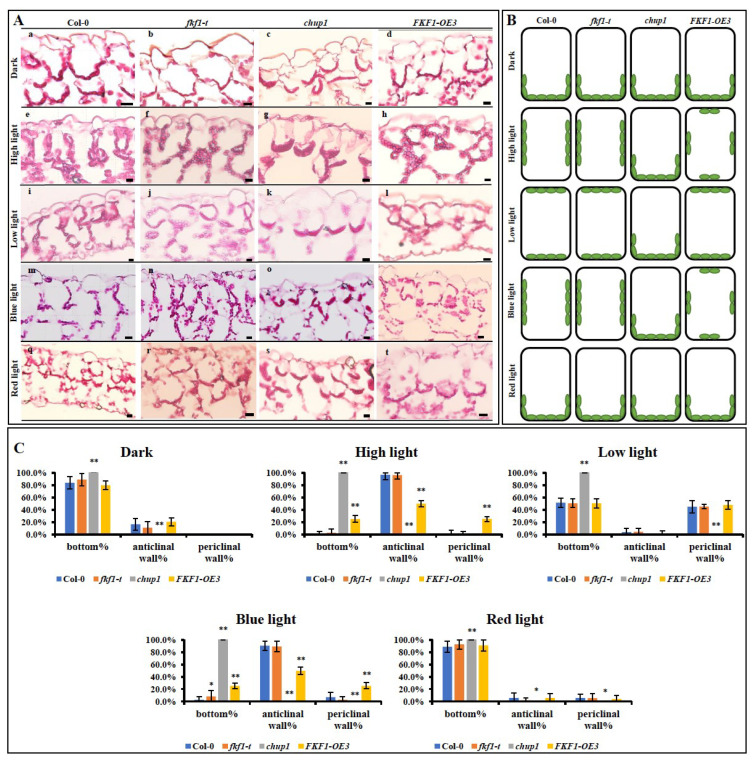
Analysis of photorelocation of chloroplasts in the palisade cells under various light conditions. (**A**) Leaf cross-sectioning analysis shows that the high light-induced alignment of chloroplasts on the anticlinal wall was compromised in the FKF1 overexpression line. The Arabidopsis lines (Col-0, fkf1, chup1, and FKF1 overexpression line) were grown under long-day white light conditions for three weeks and then were transferred to dark, high white light (120 µmol/m^2^/s), low white light (10 µmol/m^2^/s), monochromatic blue light (25 µmol/m^2^/s), or monochromatic red light (25 µmol/m^2^/s) condition for 6 h after dawn. Leaf samples were collected and used for paraffin-embedded cross-sectioning. The embedded samples were cut in 5 µm sections with microtome and stained with 0.026% (w/v) methylene blue and basic fuchsin. The stained chloroplasts were imaged and photographed with an Olympus BH2TM fluorescent microscope. The experiment was repeated three times. One representative image for each condition/genotype is shown. Scale bar: 20 µm. (**B**) Diagram corresponding to the positions of chloroplasts in different Arabidopsis lines under different light conditions in panel A. (**C**) Quantitative analysis of the chloroplast position in the palisade cells. The data are an average of 15 cells from three different plants. Error bars represent S.D. (*n* = 15). Statistical significance was determined by one-way ANOVA, followed by Dunnett multiple comparisons. Significance between Col-0 and other Arabidopsis lines under same light conditions are labeled with * (*p* < 0.05) or ** (*p* < 0.01). Y-axes: % chloroplast.

The well-established function of the CHUP1 protein is the regulation of chloroplast movement along the plasma membrane [7], and loss-of-function mutations in the CHUP1 gene result in the complete abolition of chloroplast movement [31]. Since FKF1 interacts with CHUP1, we hypothesized that FKF1 is involved in the regulation of chloroplast movement. To test this, we generated two overexpression (OE) lines of FKF1 and acquired a previously characterized T-DNA insertion mutant of FKF1 (SALK_059480c) from ABRC (Arabidopsis Biological Resource Center) [1,3,58]. Real-time quantitative PCR (qPCR) analysis showed that the expression level of FKF is significantly higher in two OE lines (OE1 and OE3) compared to Col-0 (Figure S3). We first investigated the chloroplast distribution patterns in the palisade cells under various light conditions using Col-0, *fkf1-t*, *chup1*, and the two confirmed FKF1-OE lines. Three-week-old Arabidopsis plants grown under long-day conditions were transferred to dark, high light (120 µmol/m^2^/s), or low light (10 µmol/m^2^/s) conditions for 6 h, and leaf samples then were collected and cross-sectioned. Under the dark condition, chloroplasts accumulated at the bottom of the palisade cells in Col-0, *fkf1-t*, *chup1*, and FKF1-OE lines (Figure 2A(a–d)). Under high light (~120 µmol/m^2^/s), chloroplasts accumulated at the anticlinal walls in Col-0 and *fkf1-t* cells, while chloroplasts were distributed along the plasma membrane all around the cell in the FKF1-OE line (Figure 2A(e–h)). However, the chloroplasts showed no response to high light and remained at the bottom of the palisade cells in *chup1* mutants (Figure 2A(g)). Under low light, the cross-sectioning analysis clearly showed that most chloroplasts accumulated at the periclinal walls in the Col-0, *fkf1-t* and FKF1-OE cells. As expected, in the *chup1* cells, the chloroplasts accumulated at the bottom of the palisade cells (Figure 2A(i–l)). The cross-sectioning results showed that excess levels of FKF1 protein interfered with the positioning of chloroplasts under high light (avoidance response). As a result, chloroplasts accumulated randomly in the palisade cells, instead of showing an avoidance response. This data clearly establishes the role of FKF1 in the regulation of the avoidance response.

The accumulation and avoidance movements were only induced by blue light, but not by red light, in Arabidopsis [25,29]. The two blue light receptors, phot1 and phot2, are involved in the blue light mediated chloroplast photorelocation [9,10,11]. Since FKF1could potentially be involved in the avoidance response, we investigated the distribution patterns of chloroplasts under monochromatic blue light and red light in different lines of Arabidopsis. Three-week-old Arabidopsis plants grown under long-day conditions were transferred to monochromatic blue-light or red-light conditions for 6 h. The leaf cross-sectioning results showed that monochromatic blue light caused the movement of chloroplasts toward the anticlinal walls in the Col-0 and *fkf1-t* cells (Figure 2A(m,n)), while chloroplasts in the FKF1-OE line were distributed on both the anticlinal and periclinal walls (Figure 2A(p)). In contrast, when Col-0, *fkf1-t*, *chup1*, and FKF1-OE plants were treated with monochromatic red light, most of the chloroplasts were aligned at the bottom of the palisade cells in all the lines (Figure 2A(q–t)).

The distribution patterns of chloroplasts in the palisade cells were quantitatively analyzed by counting the numbers of chloroplasts located at the anticlinal wall, periclinal wall, and cell bottom (Figure 2C). Consistent with the visual observation of the cross-sections, the quantitative data show that FKF1-OE3 exhibited a different distribution pattern of chloroplasts compared to Col-0 and *fkf1-t*. Under high light and monochromatic blue light, FKF1-OE3 showed a significantly higher number of chloroplasts located at the periclinal wall and cell bottom compared to Col-0 and *fkf1-t*, while the number of chloroplasts located at the anticlinal wall in FKF1-OE3 was significantly smaller compared to Col-0 and *fkf1-t*. These results indicate that monochromatic red light mimics the dark conditions or has no influence on chloroplast localization. In the FKF1-OE line, chloroplasts are hyposensitive to blue light or high light. Overall, these results demonstrate the role of blue light in chloroplast photorelocation through FKF1, in addition to phot1 and phot2.

### 2.4. The Chloroplast Avoidance Response to Light Is Impaired in FKF1 Overexpression Lines

**Figure 3:**
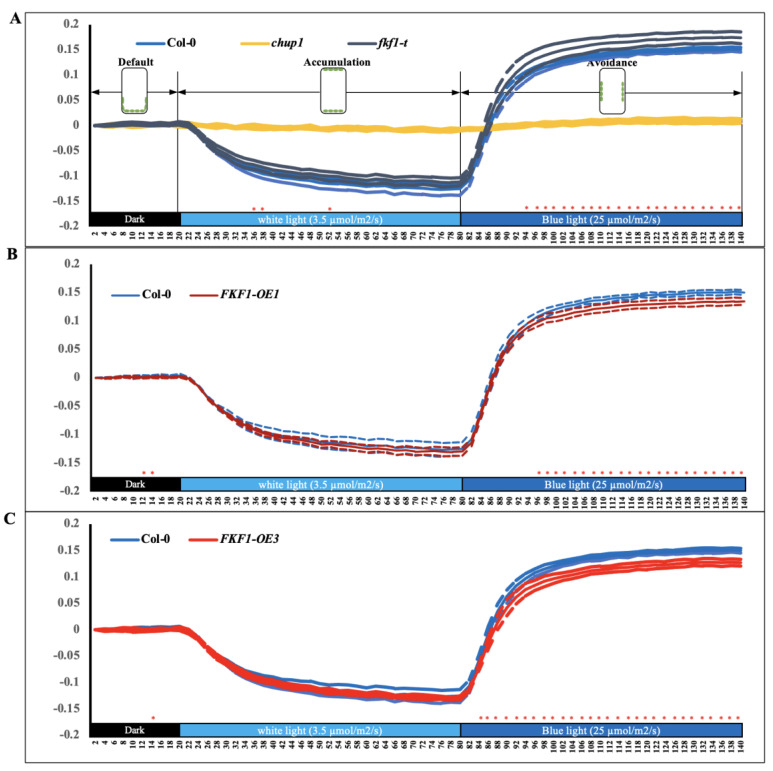
Chloroplast relocation studies using a red light transmittance assay under white light and monochromatic blue light. A red-light transmittance assay was performed to investigate the photorelocation of different Arabidopsis lines, evaluating the chloroplast movement in the Col-0, chup1, *fkf1-t*, and FKF1 OE lines in response to sequential treatments of white light and monochromatic blue light. Red-light transmittance was measured for 20 min in dark adapted leaves to establish a baseline, followed by sequential 60 min treatment of 3.5 µmol/s/m^2^ white light and 60 min treatment of 25 µmol/m^2^/s monochromatic blue light. Red-light transmittance was recorded every 2 min. The data are an average of red transmittance changes from 6 independent leaves collected from 6 different plants. Dashed lines represent S.D. (*n* = 6). Welch’s *t* test was used to test the difference between the means from two groups. Statistically significant differences between the Col-0 and any other Arabidopsis lines are labeled with * (*p* < 0.05). The diagram shows the expected positions of chloroplasts under different light conditions in Col-0. Y-axes: Δ red-light transmittance; X-axes: time in min.

Anatomical data showed that FKF1 interferes with the photorelocation of chloroplasts under high light (Figure 2). To further understand the light-induced FKF1-mediated chloroplast movement, we investigated the red-light transmittance changes in the rosette leaves of Arabidopsis, detecting chloroplast movement and determining the relative relocation speed. Transmittance changes in red light (660 nm) under different light conditions were recorded every two minutes [59]. The Col-0, *fkf1-t*, and FKF1-OE lines showed similar red-light transmittance changes in accumulation response to low light intensity (3.5 µmol/s/m^2^ white light for 60 min) (Figure 3).

In contrast, the red-light transmittance in response to monochromatic blue light (25 µmol/s/m^2^ monochromatic blue light for 60 min) was significantly higher in *fkf1-t* compared to Col-0, indicating enhanced avoidance response (Figure 3A), while the response was significantly lower in the two FKF1-OE lines compared to Col-0 (Figure 3B,C), indicating a compromised avoidance response.

**Figure 4:**
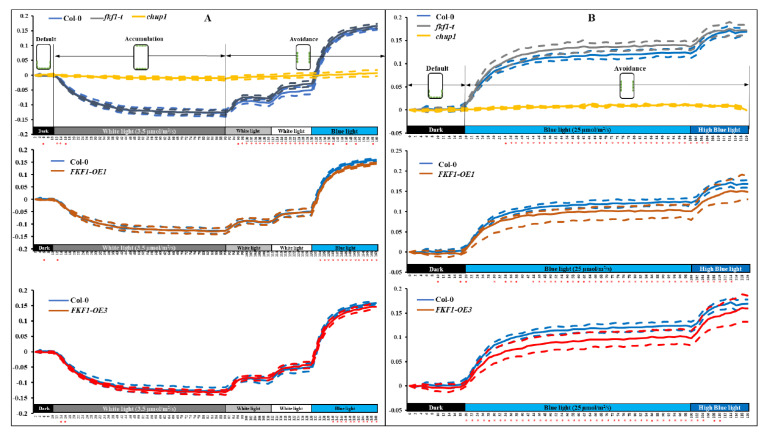
Chloroplast relocation studies using red-light transmittance assay under various light conditions. A red-light transmittance assay was performed to investigate the photorelocation of chloroplasts under various light conditions. (**A**) Chloroplast movement in Col-0, chup1, *fkf1-t*, and FKF1 OE lines in response to sequential treatments of increasing fluence rates of white light and monochromatic blue light. Red-light transmittance was measured for 10 min in dark adapted leaves to establish a baseline, followed by a sequential 80 min treatment of 3.5 µmol/s/m^2^ white light, 20 min treatment of 50 µmol/s/m^2^ white light, 20 min treatment of 100 µmol/m^2^/s white light, and finally, 30 min treatment of 25 µmol/m^2^/s monochromatic blue light. Red-light transmittance was recorded every 2 min. (**B**) Chloroplast movement in the Col-0, chup1, *fkf1-t*, and FKF1 OE lines in response to sequential treatments of increasing fluence rates of monochromatic blue light. Red-light transmittance was measured for 20 min in dark adapted leaves to establish a baseline, followed by a sequential 80 min treatment of 25 µmol/m^2^/s monochromatic blue light, and 20 min treatment of 60 µmol/m^2^/s monochromatic blue light. Red-light transmittance was recorded every 2 min. The data are an average of red-light transmittance changes from 8 independent leaves collected from 8 different plants. Dashed lines represent S.D. (*n* = 8). Welch’s *t* test was used to test the difference between the means from two groups. Statistically significant differences between the Col-0 and any other Arabidopsis lines are labeled with * (*p* < 0.05). The diagram shows the expected positions of chloroplasts under different light conditions in Col-0. Y-axes: Δ red-light transmittance; X-axes: time in min.

To obtain more detailed information on chloroplast movements in real time, we measured the red-light transmittance under 3.5 µmol/s/m^2^ white light, 50 µmol/s/m^2^ white light, 100 µmol/s/m^2^ white light, 25 µmol/s/m^2^ monochromatic blue light, and 60 µmol/s/m^2^ monochromatic blue light (Figure 4). Again, the Col-0, *fkf1-t* and FKF1-OE lines did not show differences at the accumulation stage under low light intensity (3.5 µmol/s/m^2^ white light for 80 min) (Figure 4A). In contrast, the red-light transmittance in response to high intensity white light (50 µmol/s/m^2^ white light for 20 min, followed by 100 µmol/s/m^2^ white light for 20 min) was significantly higher in *fkf1-t* compared to Col-0 (Figure 4A, upper panel). Avoidance responses of FKF1-OE lines exposed to 25 µmol/s/m^2^ monochromatic blue light were impaired, as significantly lower red-light transmittance was observed in the two FKF1-OE lines relative to Col-0 (Figure 4A, middle and lower panel).

We then tested the avoidance response under two monochromatic blue light intensities (25 µmol/s/m^2^ and 60 µmol/s/m^2^). As shown in Figure 4B, the red-light transmittance changes in response to 25 µmol/s/m^2^ were significantly higher in *fkf1-t*, but significantly lower in the FKF1-OE lines compared to Col-0. While under high monochromatic blue light (60 µmol/s/m^2^), the transmittance changes showed no significant difference between the Col-0, f*kf1*, and FKF1-OE lines. These results suggest that FKF1 negatively regulates the avoidance response under high light intensity, as the FKF1-OE lines showed fewer chloroplasts on the anticlinal wall and lower red-light transmittance than Col-0 under high light conditions (upper panels of Figure 4A,B). In contrast, the absence of FKF1 enhanced the avoidance response, as *fkf1-t* showed a higher red-light transmittance value, indicating that more chloroplasts moved to the anticlinal wall in the palisade leaves of *fkf1-t* than in Col-0. High blue light resulted in a complete failure of accumulation and avoidance responses in the *chup1* mutant (Figure 3A), in contrast to the light induced chloroplast movements observed in the FKF1 and FKF1-OE lines. Together, the leaf cross-sectioning experiment and red-light transmittance assays show that FKF1 functions as a negative regulator of the avoidance response.

### 2.5. FKF1 Regulates CHUP1 Protein Abundance

**Figure 5:**
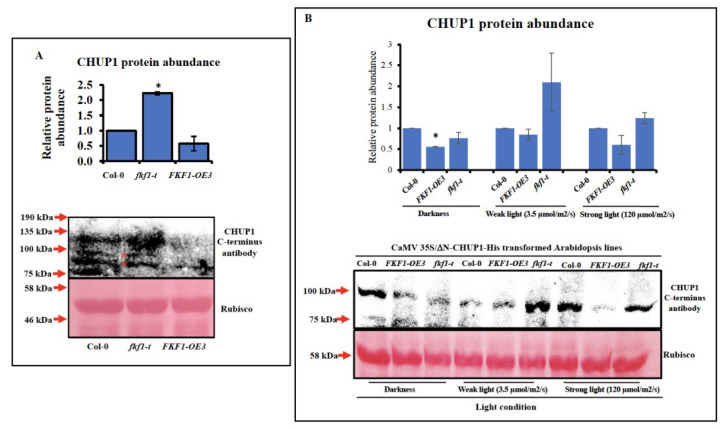
CHUP1 protein expression analysis. (**A**) Leaf samples of three-week-old Arabidopsis plants grown under long-day conditions (16 h light/8 h dark) were collected 12 h after dawn and used for Western blot analysis. The relative protein levels of CHUP1 were quantified using Ponceau S stained Rubisco large subunit (53 kDa) as an internal reference. The CHUP1 protein abundance of Col-0 was arbitrarily set as 1. The data are an average of two independent biological replicates. Error bars represent S.D. (*n* = 2). One-way ANOVA, followed by Tukey and Dunnett multiple comparisons, was used to determine the statistically significant difference between Col-0 and other Arabidopsis lines under same light conditions and are labeled with * (*p* < 0.05). Y-axis: % chloroplast. (**B**) Leaf samples of three-week-old Arabidopsis plants grown under long-day conditions (16 h light/8 h dark) were transferred to different light conditions, collected 12 h after dawn, and used for Western blot analysis. The relative protein levels of CHUP1 were quantified using Ponceau S stained Rubisco large subunit (53 kDa) as an internal reference. A 200 µg total protein sample was used for each line. The CHUP1 protein abundance of Col−0 of each light condition was arbitrarily set as 1. The data are an average of two independent biological replicates. Error bars represent S.D. (*n* = 2). One-way ANOVA followed, by Tukey and Dunnett multiple comparisons, was used to determine the statistically significant difference between Col−0 and other Arabidopsis lines under the same light conditions; they are labeled with * (*p* < 0.05). Y-axes: % chloroplast. * Represent significance.

FKF1 regulates protein function by inducing the degradation [2,60] or by preventing the dimerization [61] of its target proteins, and both mechanisms involve physical interactions with the target proteins. FKF1 showed physical interaction with CHUP1; however, the consequence of FKF1 interaction with CHUP1 protein is not known. To this end, we investigated the protein abundance of CHUP1 in three-week-old Col-0, *fkf1-t* and FKF1-OE3 plants using a CHUP1 antibody. Western blot analysis showed that the protein abundance of CHUP1 is significantly higher in *fkf1-t* than that in Col-0 and FKF1-OE3 (Figure 5A). Since CHUP1 is a large membrane anchored protein, the extraction of CHUP1 is challenging, and the Western blots showed high background noise. To overcome these difficulties, we removed the N-terminus transmembrane domain of CHUP1 and tagged the C terminus of CHUP1 with 6×His, creating a ΔN-CHUP1-6×His protein that should be expressed in the cytosol, facilitating protein extraction and Western blot analysis. Because FKF1 interacts with the C-terminus of CHUP1, we reasoned FKF1 should still interact with and induce the degradation of the truncated CHUP1 protein. The CaMV35S/ΔN-CHUP1 construct was tagged with 6×His as a backup option in case of the disruption of CHUP1 antibody specificity towards ΔN-CHUP1 (Figure 5B). Since the 6×His tag did not alter the specificity of ΔN-CHUP1, the CHUP1 antibodies were used for Western blot analysis. To compare the protein abundance of CHUP1 between Col-0, *fkf1-t* and FKF1-OE under different light conditions, three-week-old CaMV35S/ΔN-CHUP1-6×His transformed Arabidopsis plants were incubated under dark, weak white light (3.5 µmol/m2/s), or strong white light (120 µmol/m2/s) for 12 h after dawn. The light treated leaf samples were collected and used for Western blot analysis (Figure 5B). The ΔN-CHUP1 protein was more abundant in *fkf1-t* than in Col-0 and FKF1-OE3 under three different light conditions, while it was less abundant in FKF1-OE3, especially under high light. Taken together, the Western blot results suggest that the FKF1 regulates CHUP1 protein abundance through an unknown mechanism. Full-length gels are presented in Supplementary Figure S7.

### 2.6. FKF1 Alters the Expressions of Chloroplast Photorelocation Pathway Genes

**Figure 6:**
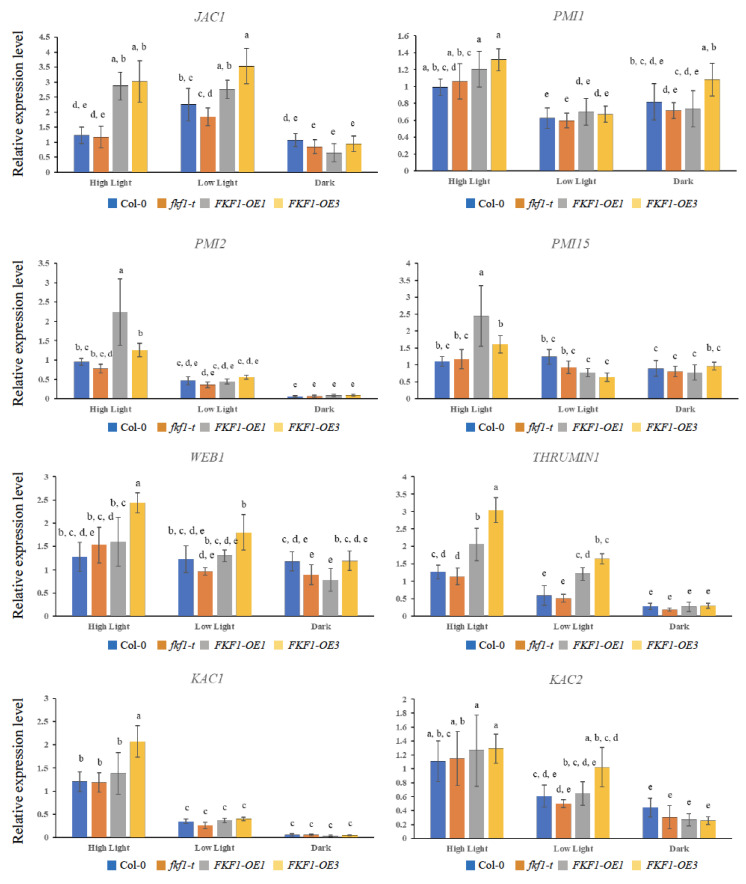
Expression analyses of critical genes involved in the chloroplast photorelocation under white light. Arabidopsis plants grown under white light for 3 weeks were transferred to high light (120 µmol/m^2^/s), low light (10 µmol/m^2^/s), or dark for 6 h (6 h after dawn). Leaf samples were then collected and used for qPCR analysis. The data are an average of two technical replicates from three biological replicates. Error bars represent S.D. (*n* = 3). One-way ANOVA, followed by post-hoc analysis using Tukey’s test, was used to determine the statistically significant differences between Arabidopsis lines. Means not sharing the same letter are significantly different (*p* < 0.05) or highly significantly different (*p* < 0.01). Actin2 was used as a reference gene.

Phot1 and phot2 are involved in the regulation of chloroplast movement. Several downstream players involved in signal transduction and cp-actin regulation have been identified, including PMI2, PMI15, PMI1, JAC1, WEB1, THRUMIN1, and two kinesin-like proteins KAC1 and KAC2 [32,33,34,35,36,37,44]. To understand the role of FKF1 in photorelocation, we measured the expression of CHUP1 along with genes related to signal transduction and cp-actin motility (*PMI2*, *PMI15*, *PMI1*, *JAC1*, *WEB1*, *THRUMIN1*, *KAC1*, and *KAC2*) in *fkf1-t* and FKF1-OE plants under different light conditions. The expression of CHUP1 was significantly induced in FKF1-OE lines only under low light (10 µmol/m^2^/s) conditions (Figure S4). The qPCR analyses revealed that *JAC1* and *THRUMIN1* were significantly upregulated in the FKF1-OE lines compared to Col-0 and *fkf1-t* under high light (120 µmol/m^2^/s) and low light (10 µmol/m^2^/s) conditions, while no significant difference was observed under dark condition among Col-0, *fkf1-t*, and OE lines (Figure 6).

**Figure 7:**
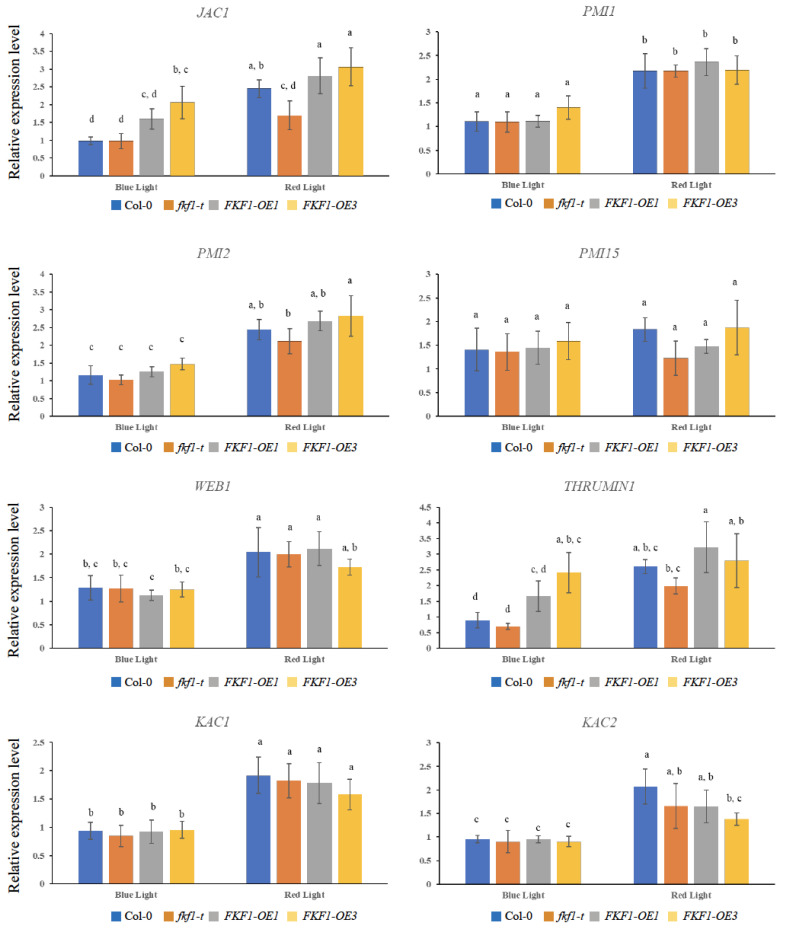
Expression analyses of critical genes involved in the chloroplast photorelocation under monochromatic red or monochromatic blue light. Arabidopsis plants grown under white light for 3 weeks were transferred to monochromatic blue light (25 µmol/m^2^/s) or monochromatic red light (25 µmol/m^2^/s) for 6 h (6 h after dawn). Leaf samples were then collected and used for qPCR analysis. The data are an average of two technical replicates from three biological replicates. Error bars represent S.D. (*n* = 3). One-way ANOVA, followed by post-hoc analysis using Tukey’s test, was used to determine the statistically significant differences between Arabidopsis lines. Means not sharing the same letter are significantly different (*p* < 0.05) or highly significantly different (*p* < 0.01). Actin2 was used as a reference gene.

The expression analyses of the *PMI1*, *PMI2,* and *PMI15* genes revealed no significant difference between the different lines under low light or dark conditions, while *PMI2* and *PMI15* showed higher expression levels under high light in the FKF1-OE1 line (Figure 6). Although the transcript levels of *WEB1, KAC1*, and *KAC2* did not show any difference between Col-0, *fkf1-t*, FKF1-OE1, and FKF1-OE3, low light and dark conditions repressed their expression (Figure 6). The expression levels of the *PMI2, PMI15, PMI1, JAC1, WEB1, THRUMIN1, KAC1*, and *KAC2* genes were also investigated under monochromatic blue and red light. These results show significantly higher levels of *JAC1* and *THRUMIN1* in the FKF1-OE lines under monochromatic blue light, but not under monochromatic red light conditions (Figure 7).

The yeast two-hybrid assay did not show any interaction between FKF1 and JAC1 or THRUMIN1 (Figure S5). Thus, while expression of the signal transduction genes *JAC1* and *THRUMIN1* was upregulated in FKF1-OE plants, FKF1 does not physically interact with JAC1 and THRUMIN1.

### 2.7. Altering the Expression Levels of THRUMIN1 and JAC1 Did Not Rescue the Impaired Avoidance Response of FKF1-OE Plant

**Figure 8:**
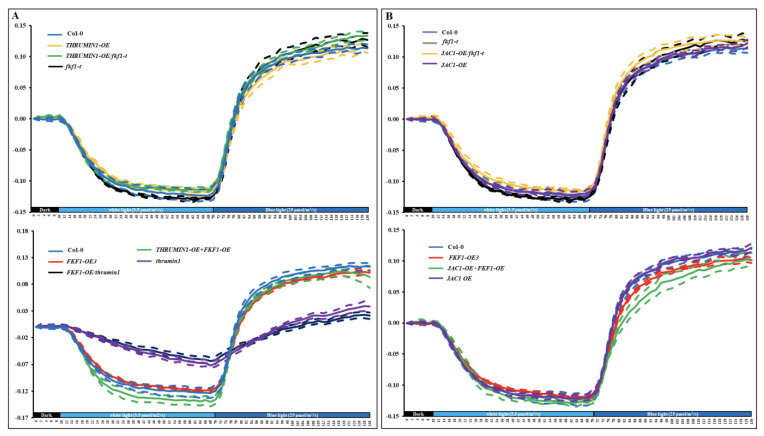
Chloroplast relocation studies using red light transmittance assay in various *Arabidopsis* lines. (**A**) Chloroplast movement in Col-0, *fkf1-t*, and FKF1-OE3, THRUMIN1-OE, *thrumin1*, THRUMIN1-OE/*fkf1-t*, FKF1-OE/*thrumin1*, and Thrumin1-OE + FKF1-OE in response to sequential treatments of white light and monochromatic blue light. Red-light transmittance was measured for 10 min in dark adapted leaves to establish a baseline, followed by a sequential 60 min treatment of 3.5 µmol/m^2^/s white light, and then a 50 min treatment of 25 µmol/m^2^/s monochromatic blue light. Red-light transmittance was recorded every 2 min. (**B**) Chloroplast movement in Col-0, *fkf1-t*, FKF1-OE3, JAC1-OE/*fkf1-t*, JAC1-OE, JAC1-OE + FKF1-OE in response to sequential treatments of white light and monochromatic blue light. Red-light transmittance was measured for 10 min in dark adapted leaves to establish a baseline, followed by a sequential 60 min treatment of 3.5 µmol/m^2^/s white light, and then a 50 min treatment of 25 µmol/m^2^/s monochromatic blue light. Red-light transmittance was recorded every 2 min. The data are an average of red-light transmittance changes from at least 3 independent leaves collected from 3 different plants. Dashed lines represent S.D. (3 ≤ *n* ≤ 8). A Student’s *t* test was used to test the difference between the means from two groups. Y-axes: Δ red-light transmittance; X-axes: time in min.

JAC and THRUMIN1 are involved in the photorelocation pathway, and both proteins showed higher gene expression levels in the FKF1-OE line under weak and high light conditions. To further understand the role of FKF1 in JAC1 and THRUMIN1 mediated photorelocation, we generated Arabidopsis Col-0 and *fkf1-t* mutant lines over expressing *JAC* and *THRUMIN1* and conducted the red-light transmittance experiment for accumulation and avoidance responses (Figure 8 and Supplementary Table S1).

Col-0 plants over expressing *THRUMIN1* showed no difference in either the accumulation or avoidance responses compared to Col-0. Over-expression of *THRUMIN1* in *fkf1-t* (THRUMIN1-OE/*fkf1-t*) showed an enhanced avoidance response similar to *fkf1-t*, and this enhanced avoidance phenotype response is likely caused by the loss-of-function of FKF1 in the *THRUMIN1* overexpression line plants. A *THRUMIN1* and *FKF1* double overexpression line (THRUMIN1-OE+FKF1-OE) showed delayed avoidance responses similar to the FKF1-OE line; in contrast, overexpression of *FKF1* in *THRUMIN1* mutants exhibited further delayed accumulation and avoidance response compared to *THRUMIN1* plants. Taken together, the red-light transmittance data show that FKF1 and THRUMIN1 are more likely to be involved in the same pathway regulating the photorelocation responses. Furthermore, Col-0 plants overexpressing *JAC1* did not show any difference in accumulation and avoidance responses compared to Col-0. Overexpression of *JAC1* in *fkf1-t* mutant showed enhanced avoidance responses similar to *fkf1-t*, while overexpression of *JAC1* in FKF1-OE3 did not rescue the impaired avoidance response of FKF1-OE3. Together, the results suggest that JAC1 and FKF1 do not participate in the same light signaling pathway regulating chloroplast movement. JAC1 appears to be a component in the accumulation response regulated by phot1 and Phot 2, while FKF1 regulates the avoidance pathway.

## 3. Discussion

Maximizing the photosynthetic efficiency under low light and minimizing the photodamage under high light through the intracellular distribution of chloroplasts is a highly controlled process, conferring on photosynthetic organisms the ability to respond to changing ambient light conditions [27]. Upon the perception of light variations by the photoreceptors, the photorelocation mechanisms are activated to physically relocate the chloroplasts. Three types of chloroplast photorelocation mechanisms operate in the cells: the accumulation response, the avoidance response, and a dark position (at the bottom of cell). The chloroplasts are moved to the periclinal walls of the palisade cells in response to low light (accumulation to maximize photosynthesis), moved to the anticlinal walls (avoidance to protect plant from photodamage), or moved to the bottom of the palisade cells under dark conditions [6]. In Arabidopsis, the cp-actin mediated chloroplast motility system is composed of cp-actin, CHUP1, THRUMIN1, and chloroplasts. The N-terminal domain of CHUP1 binds to the outer envelope of the chloroplast, allowing it to control the movement of the chloroplast [31]. The FABR domain of CHUP1 binds to the cp-actin in the cytosol, providing the traction force for the photorelocation movement [41]. The acylated N-terminal of THRUMIN1 is required for actin-dependent filament localization at the plasma membrane, whereas the Cys-rich domain is necessary for chloroplast movement [32]. It is also hypothesized that the CHUP1 protein binds to an unknown protein in the plasma membrane to facilitate the movement of the chloroplast-CHUP1 complex along the plasma membrane [7]; however, there is no experimental evidence to prove this hypothesis. The light intensity-based chloroplast movement mechanism appears to be conserved across the plant kingdom, and components of the FKF1-CHUP1 mediated chloroplast movement were discovered in cryptogams. Three Arabidopsis CHUP1 orthologues (PpCHUP1A, PpCHUP1B, and PpCHUP1C) were identified in *Physcomitrella patens*, and PpCHUP1A was proven to mediate the actin-depended light induced avoidance movements [42]. Further, an ortholog of Arabidopsis FKF1 was identified and characterized as a key protein responsible for developmental phase transition [62], similar to the flowering time mechanism in Arabidopsis. Taken together, it is tempting to speculate that the FKF1-mediated chloroplast movement mechanism is ancient and conserved across the plant kingdom, including cryptogams.

Variations in the light intensities are perceived by the photoreceptors and transmitted to the cp-actin based motility system. Phototropins (phot1 and phot2) are involved in regulating the photorelocation movement of chloroplasts [9,10,11]; however, the exact mechanism leading up to the cp-actin based movement of chloroplasts is not known. The mechanisms of phot1 or phot2 mediated photorelocation have been partially revealed; however, no direct physical interaction of phot1 or phot2 with any of the proteins involved in the photorelocation signal transduction pathway or cp-actin based motility system has been identified. Discovery of a physical interaction of FKF1 with the chloroplast-anchored protein CHUP1 has established a direct relationship between the light perception and cp-actin chloroplast motility system. The accumulation and avoidance responses appear to be regulated by different signaling mechanisms [29,30,63], as the velocity of the avoidance response varies from 1 to 2 µm/min, depending on the intensity of blue light and the abundance of the phot2 protein [63], while the velocity of the accumulation response is constant (~1 µm/min in Arabidopsis) under various light conditions [30,64]. Previous studies also suggested that phot1 and phot2 together modulate the accumulation response [9]; however, phot2 is the primary phototropin regulating the avoidance response [10,11]. A recent study suggested that phot1 and phot2 form both homocomplexes and heterocomplexes, instead of competing with each other, in fine-tuning the responses to blue light [65]. This study also showed that the *phot1phot2* double mutant did not show any movements triggered by blue light pulses, further supporting phot1 and phot2 as the two main blue light receptors controlling the signaling pathway of chloroplast photorelocation.

Both cross-sectioning and red light transmittance data showed that the accumulation response did show any difference in Arabidopsis lines including *fkf1-t*, however, the avoidance response was more intensive in *fkf1-t* but weaker in FKF1-OE lines compared to Col-0 (Figure 2 and Figure 3). These results suggest that FKF1 is directly involved in negatively regulating the avoidance response. The avoidance response in *fkf1-t* mutant or in FKF1-OE lines was different from WT, indicating that FKF1 is a factor fine-tuning the avoidance response. The inconsistency between the expression level of CHUP1 and the distribution of chloroplasts in FKF1-OE lines and Col-0 suggests that FKF1 does not regulate the movement of chloroplasts by regulating the transcription of CHUP1 (Figure 2, Figure 3 and Figure S4). On the other hand, the results of Y2H and Western blot analyses (Figure 1 and Figure 5) indicates that FKF1 regulates the chloroplast movement through regulating the abundance of CHUP1 protein. However, the transcripts of JAC1 and THRUMIN1 increased significantly in FKF1-OE lines especially under high light. We hypothesize that, at least for THRUMIN1 which is required for both accumulation and avoidance responses [32], it may also be involved in the FKF1 regulated avoidance pathway, but further research is required to reveal a specifical role.

## 4. Materials and Methods

### 4.1. Plant Growth and Light Conditions

*Arabidopsis thaliana* lines used in this study were in the Columbia-0 genetic background (Supplementary Table S1). Arabidopsis plants were grown in soil under long-day conditions (16 h light/8 h dark) at 22 °C Day/22 °C dark in growth chambers (Percival^TM^, Perry, Iowa, USA), with a light density of 120 µmol/s/m^2^ (fluorescence). The LumiGrow Pro 650 LED lighting system (LumiGrow, Emeryville, CA, USA) was used to supply white, monochromatic blue (450 nm), or monochromatic red light (650 nm). An Extech LED light meter LT45 (Extech, Nashua, NH, USA) was used to measure the light intensity.

### 4.2. Quantitative Analysis of Gene Expression

Total RNA was extracted from 100 mg plant samples using the Spectrum^TM^ Plant Total RNA kit (Sigma-Aldrich, St. Louis, MO, USA). Genomic DNA was removed from the RNA samples with the On-Column DNase I (Sigma-Aldrich) treatment. First strand cDNA was synthesized using 0.5–1 µg DNA-free RNA with a iScript^TM^ reverse transcription supermix RT-qPCR kit (Bio-Rad, Hercules, CA, USA). The resulting cDNA was diluted to 0.06 times with water and used for qPCR with the FastStart Essential DNA Green Master kit (Roche, Branchburg, NJ, USA). A LightCycler 96 (Roche, Branchburg, NJ, USA) was used to perform qPCR experiments and analyze the q-PCR results. Gene expression was calculated using the ΔΔCt method [66]. The primers used for qPCR analyses of different genes are all listed in Supplementary Table S2.

### 4.3. Plasmid Construction and Arabidopsis Transformation

The FKF1 overexpression plasmid was constructed using full-length cDNA amplified from the first strand cDNA pool using Q5 high-fidelity DNA polymerase. The forward primer used for amplification was tagged with AscI, and the reverse primer was tagged with PacI. The PCR product was then digested with AscI and PacI and subcloned into the pMDC32 binary vector digested with same set of restriction enzymes. The hygromycin resistance gene (encoding aminoglycoside phosphotransferase) was used as a selectable marker in pMDC32 for transgenic plants. The same strategy was used to generate THRUMIN1 and JAC1 overexpression plasmid constructs. AscI and PacI digested PCR produces were subcloned into the binary vector pMDC123, which contains an herbicide (Basta) resistance gene (encoding phosphinothricin acetyltransferase) as a selectable marker. The CaMV35S driven N-terminus truncated CHUP1-6×His (C-terminal tagged) construct was generated by amplification of the partial CHUP1 (without N terminus) cDNA fragment (from 76 bp–3009 bp) from the first strand cDNA pool using Q5 high-fidelity DNA polymerase. The forward primer tagged with AscI and the reverse primer with 6×His encoding sequence GTGATGGTGATGGTGATG (inserted immediately before the stop codon) was tagged with PacI. The PCR products were then digested with AscI and PacI. The two digested PCR products were then subcloned into the binary vector pMDC123 digested with SbfI and PacI. The chimeric expression cassettes were then mobilized into *Agrobacterium tumefaciens* strain GV3101 by electroporation for plant transformation. *Arabidopsis thaliana* transformation was conducted according to the previously described method [59]. The primers used in plasmid construction are all listed in Supplementary Table S2.

### 4.4. Genome-Wide Yeast Two-Hybrid Screening to Identify FKF1 Interacting Proteins

Yeast two-hybrid screening was performed by Hybrigenics Services, S.A.S., Evry, France (http://www.hybrigenics-services.com). The coding sequence for full-length *A. thaliana* FKF1 (NM_105475.4) was PCR-amplified and cloned into pB29 as an N-terminal fusion to LexA (FKF1-LexA). The construct was checked by sequencing the entire insert, using it as a bait to screen a random-primed *A. thaliana* one-week old seedling cDNA library constructed into pP6. pB29 and pP6 derive from the original pBTM116 [67,68] and pGADGH [69] plasmids, respectively. A total of 90 million clones (10-fold the complexity of the library) were screened using a mating approach with YHGX13 (Y187 ade2-101::loxP-kanMX-loxP, matα) and L40ΔGal4 (MATAa) yeast strains, as previously described [70]. A total of 132 His+ colonies were selected on a medium lacking tryptophan, leucine, and histidine. The prey fragments of the positive clones were amplified by PCR and sequenced at their 5′ and 3′ junctions. The resulting sequences were used to identify the corresponding interacting proteins in the GenBank database (NCBI), using a fully automated procedure.

### 4.5. Confirmation of FKF1 and CHUP1 Interaction

The full length *FKF1* cDNA, without a stop codon, the cDNA fragments encoding the LOV domain (aa56–aa167), F-BOX (aa211–aa256), and the Kelch repeat (aa257–aa619) of FKF1 protein were amplified from a first strand cDNA pool with Q5 high-fidelity DNA polymerase (New England Biolabs (NEB), Ipswich, MA, USA), using the specific primers (Table S2). The PCR products were then digested with SpeI and PacI (NEB, Ipswich, MA, USA) and subcloned into the SpeI and PacI digested bait vector pB29 (TRP1pro/TRP1/term.-TETpro/TcR/nos-ADH1pro/LexA/ADH1term) before the LexA, respectively. The DNA fragments encoding the N-terminus (aa1–aa25), coiled-coil domain (aa61–aa276), FABR domain (aa350–aa360), pro-rich region (aa670–aa710), C-terminus (partial) (aa707–aa937), and C-terminus (aa707–aa1004) of the CHUP1 protein were amplified from a first strand cDNA pool with Q5 high-fidelity DNA polymerase. The PCR products were then digested with SfiI (New England Biolabs, U.S.A.) and subcloned into the SfiI digested prey vector pP6 (LEUpro/LEU2/term.-AmpRpro/AmpR/nos-ADH1pro/GAL4/ ADH1term) after the GAL4 activity domain, respectively. The chimeric expression cassettes were transformed into diploid yeast strain (MATAa) as a result of the mating between the two haploid strains L40deltaGal4 and Y187 (+ade2-101::loxP-kanMX-loxP) by electroporation. The primers used in plasmid construction are all listed in Supplementary Table S2.

### 4.6. Bimolecular Fluorescence Complementation (BiFC)

Bimolecular fluorescence complementation was performed as described [55]. Briefly, full length CHUP1 cDNA, without a stop codon, and full length CERK1 cDNA, without a stop codon, were amplified from the first strand cDNA pool with Q5 high-fidelity DNA polymerase. The PCR products were then digested with AscI and SwaI (for CHUP1), or AscI and KpnI (for CERK1), and subcloned into binary vector pDEST-^GW^VYCE. The full length FKF1 cDNA, without a stop codon, and the full length CERK1 cDNA, without a stop codon, were amplified from the first strand cDNA pool with Q5 high-fidelity DNA polymerase. The PCR products were then digested with AscI and KpnI (for CERK1) and subcloned into binary vector pDEST-^GW^VYNE. These chimeric binary vectors were then mobilized into *Agrobacterium tumefaciens* strain GV3101 by electroporation. Agrobacterium harboring the desired binary vector was cultivated overnight at 28 °C in liquid L.B. medium. The bacterial culture was centrifuged, resuspended, and washed with 1 mL infiltration buffer (100 mM MgCl2, 100 μM Acetosyringone) three times. The resuspended bacterial cells were then incubated in 1ml infiltration buffer at room temperature for 2 h. Agrobacterium cells were diluted to an OD600 of 0.4 and mixed. Leaves of four-week-old *Nicotiana benthamiana* were syringe-infiltrated with diluted bacterial culture, and the infiltrated plants were grown under a 16 h day/8 h dark photoperiod at 23 °C for 3–5 days. The infiltrated leaves were then examined and photographed using an Olympus BH53 fluorescent microscope equipped with a U-HGLGPS light resource and a CFP filter (excitation wavelength 426–450 nm, emission wavelength 502–538 nm). The primers used in plasmid construction are all listed in Supplementary Table S2.

### 4.7. Red-Light Transmittance Studies

The red-light transmittance experiment was performed following a previously described method, with minor modifications [59]. Briefly, 250 µL ½ MS medium with 0.5% (*w*/*v*) phytagel was poured into each well of a 96-well plate and left until it became solid. Fully expanded leaves were detached from four-week-old Arabidopsis plants 12 h after dawn and placed (adaxial side up) at the center of the 96-well plate, one leaf for each well. The plate was then sealed with a transparent film, and two small holes were made over each well, using forceps. The plate was kept in the dark at room temperature overnight. The next day, the plate was set on a DTX 880 Multimode Detector (Beckman Coulter, Brea, CA, USA) under dim red-light conditions. Transmittance changes under different light conditions were recorded every two minutes using a 660 nm measuring light. The leaf samples in the plates were irradiated with white or monochromatic blue light using the LumiGrow Pro 650 LED lighting systems (LumiGrow, Emeryville, CA, USA). For each leaf, the change of red-light transmittance was calculated as (T_o_–T_t_), where T_o_ and T_t_ are the transmittance of red-light at the first time point and other time points, respectively.

### 4.8. Histochemical Analysis of Chloroplasts Positioning

Histochemical analysis of chloroplasts positioning in the Arabidopsis rosette leaves was performed following a previously described protocol, with minor modifications [71]. Briefly, the rosette leaves were sampled and fixed in FAA (formaldehyde-acetic acid-ethanol), and subsequently dehydrated with ethanol and xylene and then embedded in paraffin blocks. The embedded samples were then cut into 5 μm sections with microtome and dried on objective slides. To stain the chloroplasts in the leaves, the sections were subsequently washed with xylene and ethanol, and then double stained with 0.026% (*w*/*v*) methylene blue and basic fuchsin. The stained chloroplasts were imaged and photographed with an Olympus BH2TM fluorescent microscope.

### 4.9. Western Blot Analysis

Western blot analysis was conducted, according to previously described methods [57]. Briefly, protein was isolated from Arabidopsis with isolation buffer (150 mM NaCl, 1% Triton X, 0.1% SDS, 50 mM pH8.0 Tris-HCl, 1 mM PMSF, 1% β-mercaptoethanol, 1 tablet of Pierce Protease Inhibitor/10 mL isolation buffer), and then precipitated with 100% Trichloroacetic acid. The protein pellet was washed with 100% ice-cold ethanol and resuspend in protein dissolving buffer (8 M Urea, 5 mM dithiothreitol). For SDS-PAGE, the protein solution containing 100 µg total protein was mixed with 5 × SDS loading buffer (10% SDS, 50% glycerol, 20% β-mercaptoethanol, 0.25 M pH 6.8 Tris-HCl, 0.02% bromophenol blue), denatured at 70 °C for 10 min, and separated on an SDS-PAGE gel and transferred to a PVDF membrane using the iBlot2 Western blot transfer system (Thermo Fisher Scientific, Waltham, MA, USA). Hybridization was performed using anti-CHUP1-C-terminus mouse monoclonal antibody as the primary antibody (Abmart, Xuhul, Shanghai, China), at a dilution of 1:3000 in 5% (*w*/*v*) BSA TBST (Tris-Buffered Saline-Tween), and HRP coupled goat anti mouse IgG as the secondary antibody, at a dilution of 1:5000 in 5% (*w*/*v*) BSA TBST (Tris-Buffered Saline-Tween). The signal was developed with Clarity Western ECL Substrate (Bio-Rad, Hercules, CA, USA) and detected using Chemidoc XRS+ imager (Bio-Rad, Hercules, CA, USA). The relative protein levels of CHUP1-His were quantified using Ponceau S stained Rubisco large subunit (53 kDa) as an internal reference.

### 4.10. Statistical Analysis

A Student’s *t* test was used to test the difference between the means from the two groups; *p* < 0.05 was considered statistically significant and marked as *; *p* < 0.01 was considered statistically highly significant and marked as **. Welch’s *t* test was used to test the difference between the means from the two groups. Post hoc comparisons using the Tukey HSD test were used to determine the statistically significant differences between the means from three or more groups. Means not sharing the same letter were considered to be statistically significantly different (*p* < 0.05) or statistically highly significantly different (*p* < 0.01).

## 5. Conclusions

**Figure 9:**
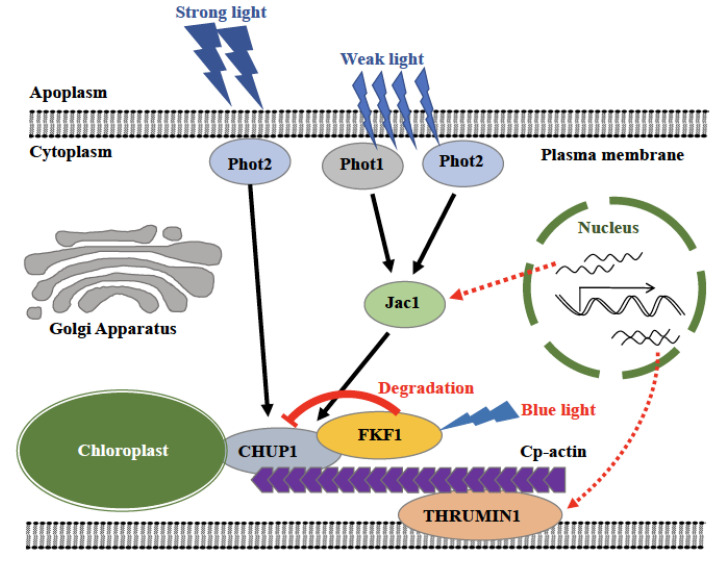
Model of FKF1 mediated photorelocation of chloroplasts. Phot1 and phot2 together perceive the low light and regulate the accumulation response through the JAC1 mediated signaling pathway, while phot2 alone perceives the high light and regulates the avoidance response through another signaling pathway. Upon light irradiate, FKF1 repress the avoidance response by regulating the CHUP1 protein abundance. The expression of JAC1 and THRUMIN1 are induced in the FKF1-OE line through an unknown mechanism.

Overall, the present study establishes a direct role of photoreceptors in the cp-actin based chloroplast photorelocation system in Arabidopsis. Here, we show that FKF1 physically interacts with CHUP1 through its C-terminus and regulates the protein stability in order to regulate the chloroplast movement. Protein stability, gene expression, avoidance and accumulation response studies suggest a novel mechanism of FKF1 mediated regulation of chloroplast movement in Arabidopsis, different from phototropins mediated chloroplast movement (Figure 9). We postulate that under low light, JAC1 perceives the signal from plasma membrane anchored phot1 and phot2 and promotes the accumulation response, while under high light, phot2 perceives the light signal to mediate the avoidance response (8–11). It is also possible that these two mechanisms operate in conjunction to maximize photosynthesis under low light and avoid photodamage under high light, hence, the absence or reduced levels of FKF1 may help in enhanced photosynthetic efficiency. Blue light is energy dense, and supplemental blue light increased the rate of photosynthesis [72] and enhanced the biomass content in lettuce and roses [73,74]; however, the underlying mechanism is not known. We hypothesize that FKF1 protein may fine tune the avoidance responses by regulating the protein level of CHUP1, which may then promote the expression of THRUMIN1, which is a critical component in the CHUP1-CP-Actin-THRUMIN1 complex and compensates for the impaired avoidance response. To precisely understand the mechanism of chloroplast movement in Arabidopsis, it is critical to further study the components of phototropins and the FKF1 pathways.

## Data Availability

Not applicable.

## Supplementary Materials

The following supporting information can be downloaded at: https://www.mdpi.com/article/10.3390/plants12030542/s1, Figure S1. Identification of FKF1 interacting proteins using high throughput Y2H screening of the *Arabidopsis thaliana* seedling library; Figure S2. Full length FKF1 did not show an interaction with full length CHUP1 protein; Figure S3. Full length FKF1 interacts with CHUP1 in planta; Figure S4. Overexpression lines showed higher levels of FKF1 transcripts; Figure S5. Expression analyses of CHUP1 under different light conditions; Figure S6. Interaction test of FKF1 between JAC1 and THRUMIN1; Figure S7. Full length gels of Figure 5; Table S1. List of overexpression and mutant lines; Table S2. List of primers used in various experiments.
