# Supplementary material for: FKF1 Interacts with CHUP1 and Regulates Chloroplast Movement in Arabidopsis
Source: Plants (Basel). 2023 Jan 25;12(3):542. doi: 10.3390/plants12030542 (PMC9920714; doi:10.3390/plants12030542)
Supplement: Supplementary file 1 [file plants-12-00542-s001.zip › plants-2142675-supplementary.pdf]

Supplementary Figures S1-S6

Supplementary Figure S1

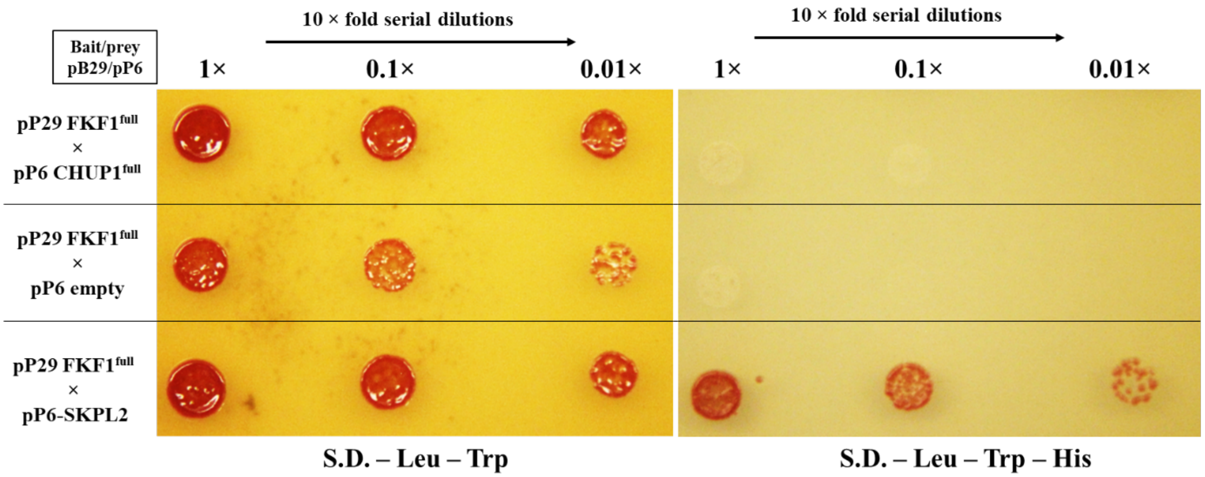

Supplementary Figure S1. Full length FKF1 did not show interaction with full length CHUP1 protein

FKF1 protein did not show interaction with and full length CHUP1 protein in yeast two hybrid assay due to the presence of transmembrane domain in CHUP1. Full length FKF1 cDNA was cloned into pB29 bait vector and full length CHUP1 cDNA was cloned in pP6 prey vector and tested for interaction. pP6 empty prey vector and pP6-SKPL2 were used as negative and positive prey vector, separately. Media: S.D. (-Leu.-Trp.) is a selection medium lacking leucine, tryptophan, and S.D. (-Leu.-Trp.–His.) is a selection medium lacking leucine, tryptophan, and histidine.

Supplementary Figure S2

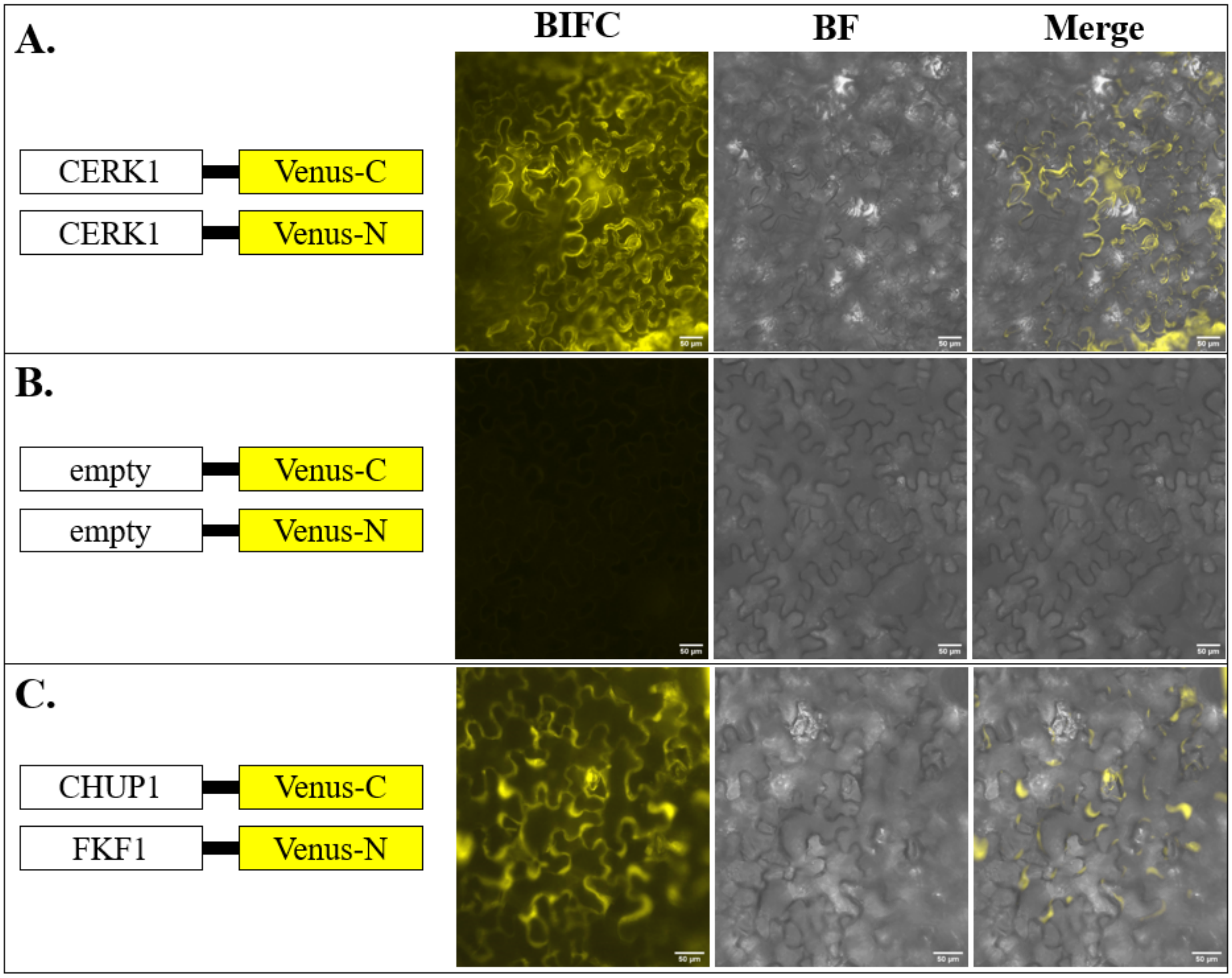

Supplementary Figure S2. Full length FKF1 interacts with CHUP1 in planta.

The schematic diagrams of the constructs used for BiFC are shown at the left of each experiment and the results are shown on the right side. Constructs were infiltrated into the tobacco leaves and images were detected three days after infiltration using Olympus BH53 fluorescent microscopy equipped with U-HGLGPS light resource and CFP filter (Excitation wavelength 426-450 nm, Emission wavelength 502-538 nm). A. Positive constructs (CERK1-VYCE and CERK1-VYNE) showed interaction. B. Negative control (Empty-VYCE and empty-VYNE) did not show interaction. C. FKF1 showed interaction with CHUP1 (CHUP1-VYCE and FKF1-VYNE). Scale bar: 50 µm.

Supplementary Figure S3

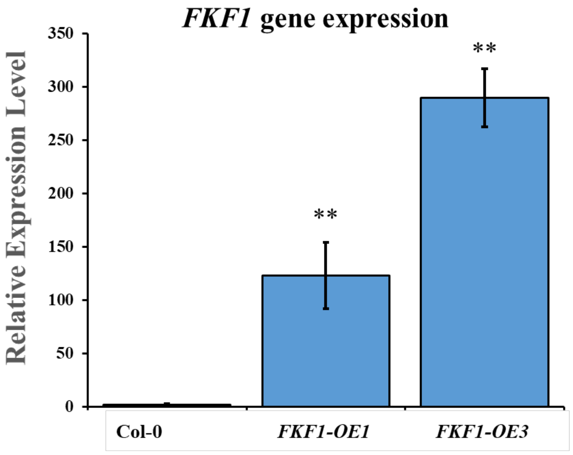

Supplementary Figure S3. Over expression lines showed higher levels of FKF1 transcripts.

The expression of FKF1 is significantly increased in overexpression lines. Leaf samples of three-week-old Arabidopsis plants (Col-0 and two FKF1-OE lines) were used for qPCR analysis. The data was an average of two technical replicates from three biological replicates. Error bars represent S.D. (n=3). Statistically significant difference is labeled with * (p<0.05)., while Statistically high significant difference is labeled with ** (p<0.01). Actin2 was used reference gene.

Supplementary Figure S4

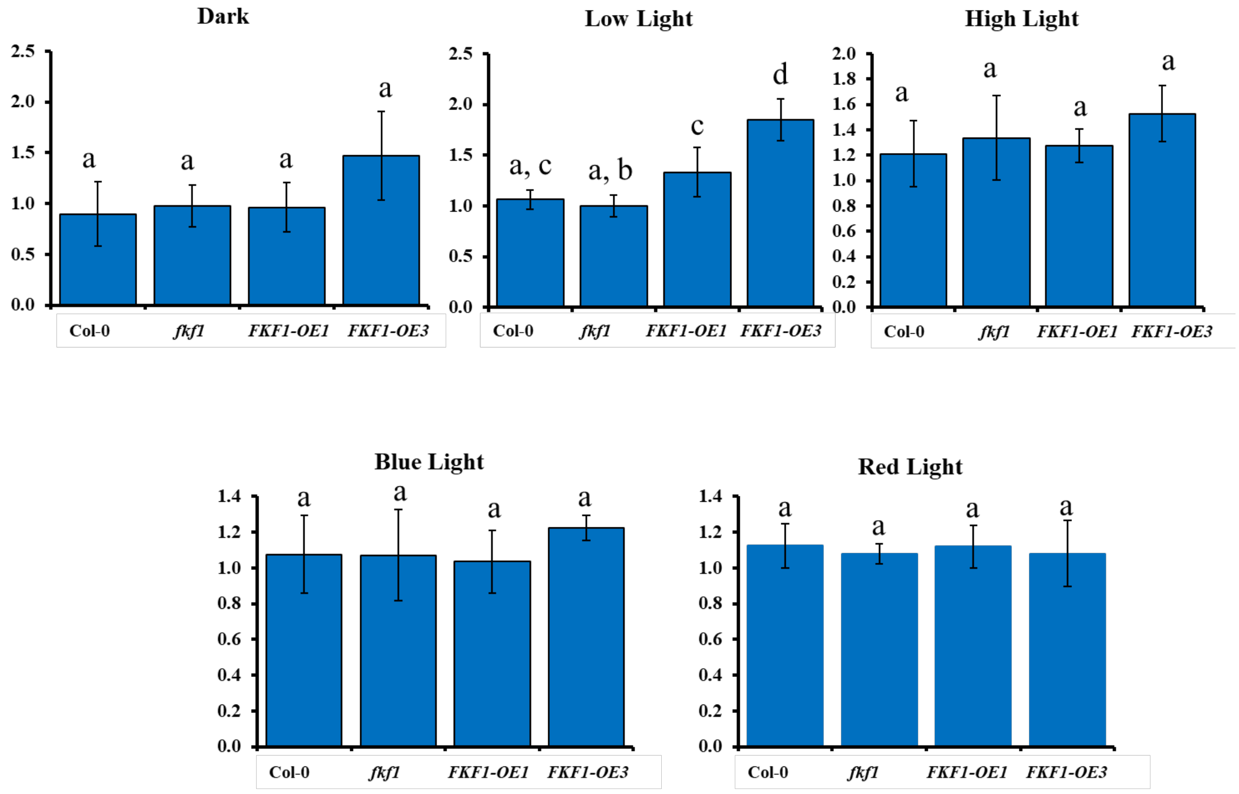

Supplementary Figure S4. Expression analyses of CHUP1 under different light conditions

The expression of CHUP1 was not significantly different between different Arabidopsis lines under various light conditions. Three-week-old white light grown Arabidopsis plants (Col-0, fkf1 and FKF1-OE lines) were transferred to high white light (120 µmol/m2/s), low white light (10 µmol/m2/s), dark, monochromatic blue light (25 µmol/m2/s), or monochromatic red light (25 µmol/m2/s) for 6 hours. Leaf samples were then collected and used for qPCR analysis. The data are an average of two technical replicates from three biological replicates. Error bars represent S.D. (n=3). Statistically significant differences between different groups was determined by one-way ANOVA. Means not sharing the same letter are significantly different ($P<0.05$) or highly significantly different ($P < 0.01$). Actin2 was used reference gene.

Supplementary Figure S5

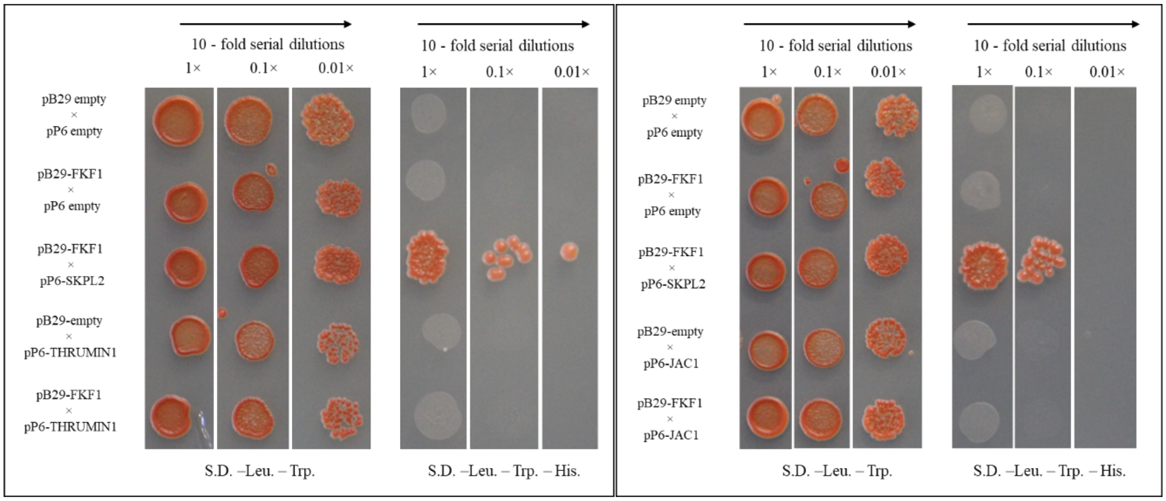

Supplementary Figure S5. Interaction test of FKF1 between JAC1 and THRUMIN1

No interaction was detected between full length FKF1 protein and full length JAC1 protein or full length THRUMIN1 protein in Yeast two Hybrid system. Full length cDNA encoding FKF1 was constructed in pB29 bait vector and used as a bait to test its interaction with THRUMIN1 or Jac1 constructed in pP6 prey vector in yeast. pP6 empty prey vector and pP6-SKPL2 were used as negative and positive prey vectors, respectively. Media: S.D. (-Leu.-Trp.) is a selection medium lacking leucine, tryptophan, and S.D. (-Leu.-Trp.–His.) is a selection medium lacking leucine, tryptophan, and histidine.

Supplementary Figure S6

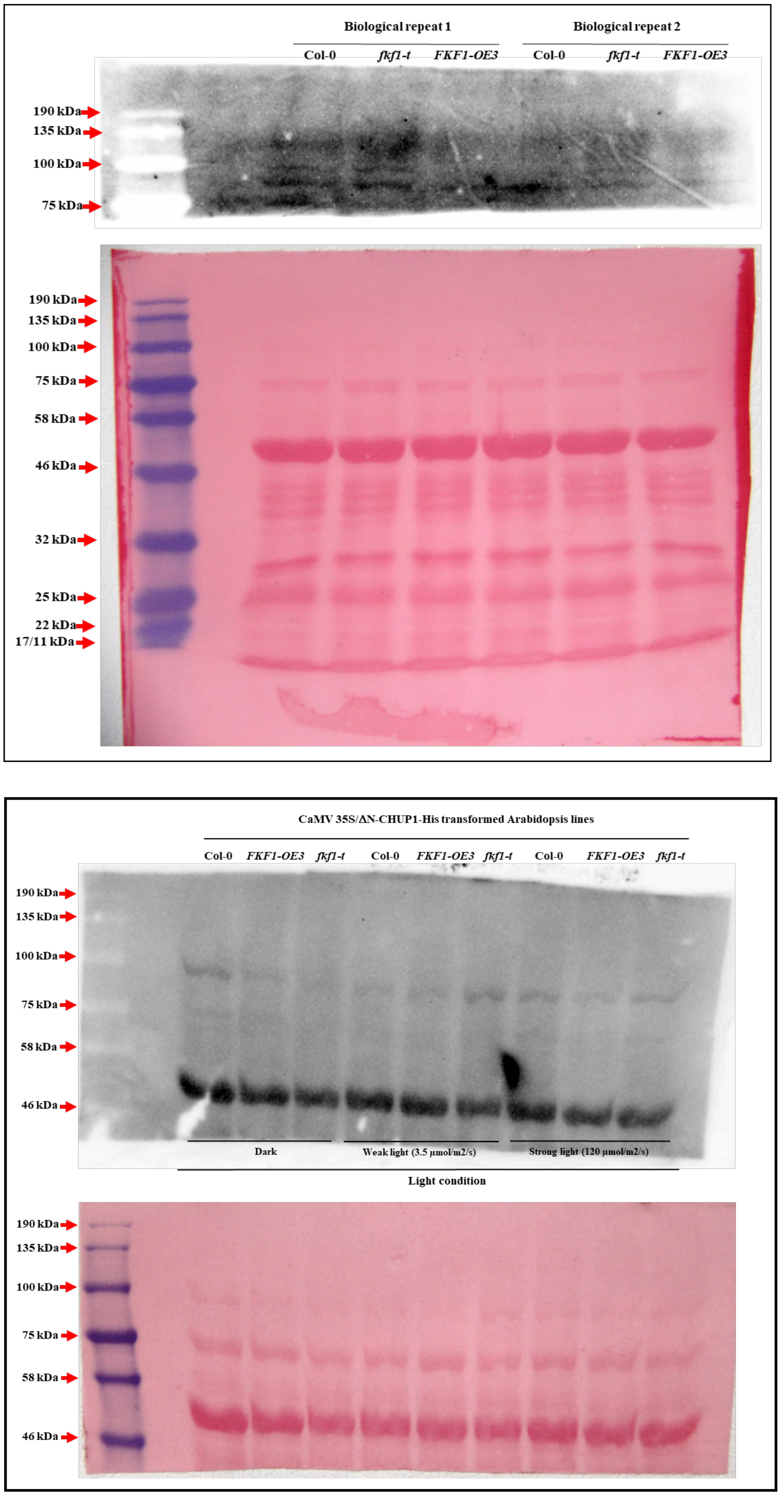

Supplementary Figure S6. Full-length gels of Figure 5.

Supplementary Tables S1 to S2

Supplemental Table S1. List of overexpression and mutant lines

Overexpression and mutant lines	
**Arabidopsis line name**	**method of generating A.th line**
A.thaliana mutant *fkf1-t*	Purchased from TAIR. T-DNA insertion mutant SALK_05948C
A.thaliana mutant *chup1*	Purchased from TAIR. T-DNA insertion mutant SALK_105043C
A.thaliana mutant *thrumin1*	Purchased from TAIR. T-DNA insertion mutant SALK_028684
FKF1 OE1/OE3	*A.thaliana* Col-0 transformed with CaMV35s driving *FKF1* gDNA
THRUMIN1 OE	*A.thaliana* Col-0 transformed with CaMV35s driving *THRUMIN1* cDNA
THRUMIN1 OE/fkf1-t	*fkf1-t* transformed with CaMV35s driving *THRUMIN1* cDNA
THRUMIN1 OE × FKF1 OE	*FKF1 OE3* transformed with CaMV35s driving *THRUMIN1* cDNA
thrumin1 × FKF1 OE3	*thrumin1* crossed with *FKF1 OE3*
JAC1 OE	*A.thaliana* Col-0 transformed with CaMV35s driving *JAC1* cDNA
JAC1 OE/fkf1-t	*fkf1-t* transformed with CaMV35s driving *JAC1* cDNA
JAC1 OE × FKF1 OE	*FKF1 OE3* transformed with CaMV35s driving *JAC1* cDNA

Supplemental Table S2. List of primers used in various experiments

**Primers**			
**Gene**		**Primer sequence**	**Notes**
FKF1	F	CTTATCTCGGTTAACTCCAAGGG	
	R	TCTCTTTTCCCCACGCATTC	
Actin	F	GCACCCTGTTCTTCTTACCG	
	R	AACCCTCGTAGATTGGCACA	
WEB1	F	CCTAGTGTCAAGATTACCGAGGCTG	
	R	GAGATGATACAGTTCTAGGTGACCCC	
THRUMIN1	F	GACAGAGAACGGAGGGAGAAAGAAG	
	R	AGACAATCAAACCTTCCCCTCCTC	
KAC1	F	GCAATTGCATCTAAGGTTAACGGA	
	R	CCTAGCCTCAGTCTCAAGGACAAAC	
KAC2	F	CAAAATCTCCAGTCTAAAGTGAAAGA	
	R	GAAGAATCGAGTGTGTTCTCAGCTG	
JAC1	F	TACCCGATAAATCCAGCTTCGCA	
	R	TTGTCCAAGAGATTCCCCACCTG	
PMI1	F	TATGGAGAAAGACGGTGGAGCTG	
	R	CGTCATTTTAGGGCTCGGAACAC	
PMI2	F	TTAGGATGCAGAGGTCGCTATCG	
	R	CGCTCAGCCTAACCGATTTTCTC	
PMI15	F	TTGGAAGAGGCGGAAAGATCGAA	
	R	TCCCCTGAACTCTCCTCTCCATT	
CHUP1	F	CCAAACCAAGCAAACCATCAG	
	R	TGTCATTGAGATTGTAGTCGGG	
**Primers for cloning of promoter regions**			
promoter		Primer sequence	Note
CHUP1 pro	F	ataCCTGCAGGCCTTTAGACATTTAGTTTT TAGGGTTTCG	SbfI tagged
	R	ataGGCGCGCCATATTAAAATCTTGAAAT GGGAATCAATTCCC	AscI tagged
**Primers for cloning of cDNA**			
Gene		Primer sequence	Note
FKF1	F	gcttGGCGCGCCcATGGCGAGAGAACATG CGATCG	AscI tagged
	R	ggccTTAATTAATTACAGATCCGAGTCTT GCCGGC	PacI tagged
CHUP1	F	ataGGCGCGCCATGTTTGTCCGGATAGGG TTTGTT	AscI tagged

	F (N terminus removed)	ataGGCGCGCCATGTCCAAACCAAGCAAACCATCAGATAATGGCG	AscI tagged
	R	gcgTTAATTAATCAGTGATGGTGATGGTGATGGTTTACAGATTCTTCTTCATT	PacI tagged 6×His tag
JAC1	F	ataGGCGCGCCAATGCAGACATTACCAAGCTCAGAA	AscI tagged
	R	gcgTTAATTAATCAAACCGGTCCGAGAGTGTTGAA	PacI tagged
THRUMIN1	F	ataGGCGCGCCATGGGGTGTACATCTTCCAAGCAAGC	AscI tagged
	R	gcgTTAATTAATCAATTAACAAAACACACGGGACAAC	PacI tagged
**Primers for BiFC analyses**			
Gene		Primer sequence	Note
CHUP1	F	ataGGCGCGCCATGTTTGTCCGGATAGGGTTTGTT	AscI tagged
	R	cgcATTTAAATccGTTTACAGATTCTTCTTCATT	SwaI tagged
FKF1	F	gcttGGCGCGCCcATGGCGAGAGAACATGCGATCG	AscI tagged
	R	aatGGTACCCAGATCCGAGTCTTGCCGGCTAGCC	KpnI tagged
CERK1	F	gcttGGCGCGCCATGAAGCTAAAGATTTCTCTAATCGC	AscI tagged
	R	aatGGTACCCCGGCCGGACATAAGACTGACTAAATC	KpnI tagged
**Primers for yeast two hybrid analyses**			
FKF1 full length (withou stop codon)	F	gactagtATGGCGAGAGAACATGCGATCGGAG	SpeI tagged
	R	attaattaaCAGATCCGAGTCTTGCCGGCTAGCC	PacI tagged
FKF1 LOV domain	F	aactagtCCAATGACTCCGCCTTCG	SpeI tagged
	R	attaattaaACGGTCAAGGTCTATAGTCGT	PacI tagged
FKF1 F-BOX domain	F	aactagtCATCATGAAGATTTCTGTGGG	SpeI tagged
	R	attaattaaCTCTTTTCCCCACGCATTCTG	PacI tagged
FKF1 Kelch domain	F	aactagtAGTGTGAGGAAGATGGTATGT	SpeI tagged
	R	attaattaaCAGATCCGAGTCTTGCCGGCTAGCC	PacI tagged
CHUP1 partial (near C- terminal)	F	catGGCCGCAGGGGCCacgaaAGCACTTGGAAGAGGAGCGGGAG	SifI tagged
	R	aggGGCCCCAGGGGCCacgaaCACTGAATCTAGCTCATAAGCTA	SifI tagged

CHUP1 N terminal	F	aGGCCGCAGGGGCCacgaaaATGTTTGTCC GGATAGGGTTTGTTG	SifI tagged
	R	cggGGCCCCAGGGGCCacgaaCTCTTGCAG ATTCTTGTCAT	SifI tagged
CHUP1 CC domain	F	catGGCCGCAGGGGCCacgaaaAAGAATCT GCAAGAGGAGGAA	SifI tagged
	R	aggGGCCCCAGGGGCCacgaaAAGCTCTCT ATTTTTTCTCTT	SifI tagged
CHUP1 FABR domain	F	catGGCCGCAGGGGCCacgaaaGATAAAGT GGCGAAAGTTAG	SifI tagged
	R	aggGGCCCCAGGGGCCacgaaTGAAGGTTG AGAATAGTTGCT	SifI tagged
CHUP1 PRR domain	F	catGGCCGCAGGGGCCacgaaaAAAAGTAC TAACTTGCCTTCA	SifI tagged
	R	aggGGCCCCAGGGGCCacgaaTTTGTTCCC GCCTCCCGCTCC	SifI tagged
CHUP1 C terminal domain	F	catGGCCGCAGGGGCCacgaaaGCACTTGGA AGAGGAGCGGGAG	SifI tagged
	R	aggGGCCCCAGGGGCCacgaaGTTTACAGA TTCTTCTTCATTG	SifI tagged
